# Implementation of the World Health Organization Age-Friendly Principles: A Case Study from Portugal

**DOI:** 10.3390/ijerph20156532

**Published:** 2023-08-05

**Authors:** Jéssica Tavares, Gonçalo Santinha, Nelson Pacheco Rocha

**Affiliations:** 1 Research Unit on Governance, Competitiveness and Public Policies (GOVCOPP), Department of Social, Political and Territorial Sciences, University of Aveiro, 3810-193 Aveiro, Portugal; 2 Institute of Electronics and Informatics Engineering of Aveiro (IEETA), Department of Medical Sciences, University of Aveiro, 3810-193 Aveiro, Portugal; npr@ua.pt

**Keywords:** ageing demographics, healthcare, healthcare administrators, age-friendly principles, WHO principles

## Abstract

Demographic ageing has emphasized the need to adapt current healthcare systems to the comorbidity profile of older adults. In 2004, the World Health Organization (WHO) developed the Age-Friendly Principles, but the approach to their implementation in the health systems still remains uncertain. This article intends to address this gap by assessing how the Principles are perceived and implemented in the Portuguese National Health Service (NHS), where this topic has recently been placed on the political agenda. A questionnaire survey was administered to primary care directors and hospital administrators, covering a total of 173 health units. Findings show that most respondents are unaware of the WHO Principles (71%) and do not identify the current organizational structure of care as a problem for the provision of care (80%). However, the implementation of the WHO Principles is lower than desired, especially regarding professional training and the management system (50% and 28% of the criteria are implemented, respectively). These criteria defined by the WHO are implemented in a reduced number of health units, as opposed to the physical environment where implementation is more widespread (64%). Accordingly, further dissemination and implementation support in the national territory are needed in order to improve the health outcomes of older adults and increase the performance of health units.

## 1. Introduction

Population ageing, resulting from declining birth rates and increasing life expectancy, has become one of the most significant challenges worldwide [1]. In 2018, the number of individuals aged 65 and older exceeded the number of those aged 0 to 4 years (705 million and 680 million individuals, respectively), indicating a growing trend of population ageing [2].

Although demographic ageing is an inevitable result of social development, it poses new challenges for health systems, health policies, and healthcare providers [3]. As individuals age, their intrinsic capacity, which is the sum of their physical and mental abilities, tends to decline, and health issues become more chronic and complex [4]. Multimorbidity, which refers to the presence of multiple chronic conditions simultaneously, is increasingly prevalent with age [5], as is the development of geriatric syndromes, including frailty [6], urinary incontinence [7], and propensity to fall [8]. This susceptibility increases the demand for healthcare by older adults and their family members [9].

However, healthcare organizations were originally designed for a relatively young population, focusing on curative care for a different set of health needs than those faced by populations today. Historically, services were structured around the diagnosis and treatment of acute health problems, predominantly communicable diseases, using a biomedical “find it and fix it” approach [10]. Today, healthcare is no longer prepared to effectively manage the healthcare needs of older adults [11].

**Figure 1:**
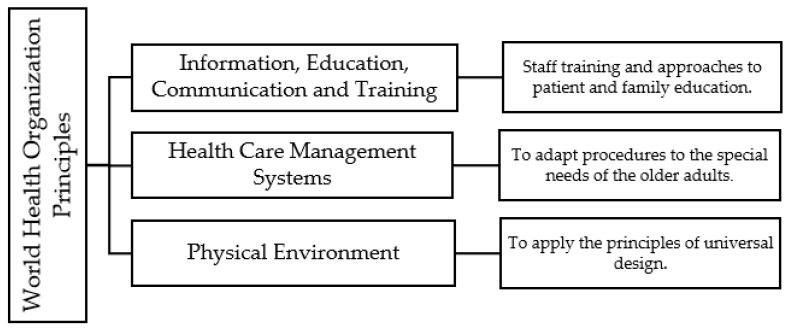
Age-Friendly Principles defined by the WHO.

Recognizing the need to develop policies and measures to ensure health promotion, disease prevention, and the provision of equitable, person-centered, high-quality care [12], the World Health Organization (WHO) formulated the Age-friendly Principles in 2004 [13]. These aim to make healthcare more aware of and responsive to the needs of older people and the type of care they require, focusing on three main areas (see Figure 1).

In 2008, the WHO developed a toolkit to support the implementation of these Principles, including several tools that can be used by healthcare professionals to assess and adapt care for older adults in primary care and hospital settings [14].

Years later, the Principles were included in the Portuguese proposal of the National Strategy for Active and Healthy Ageing 2017–2025 (NSAHA), developed by the Directorate-General of Health, proposing the generalization of the WHO project through several actions that allow its implementation [15].

However, a systematic review carried out on this topic [16] revealed that the WHO concept is being considered in world healthcare in a smaller capacity than desired. Even in the sparse European studies, it was found that none used the Principles exactly as defined by the WHO, and there were no Portuguese studies empirically analyzing this concept.

Portugal is a European country with a high rate of demographic ageing, with older age groups representing a significant part of the population [17,18]. According to Eurostat, in 2021, 22.4% of the population in Portugal was 65 or older, the third-highest figure in the European Union (20.8%) after Italy (23.5%) and Greece (22.5%) [19]. In 2020, Portugal reported a low value for healthy life years (59.7 years), below the European Union average (64 years), showing that the growth in life expectancy has not been accompanied by good health conditions [20].

Since 1979, the Portuguese health system has been centered around the Portuguese National Health Service (NHS), financed mainly through taxes. The planning and regulation of health services take place at the central level by the Ministry of Health and its institutions, and its organization is decentralized. Thus, the organization of public health services is the responsibility of the Directorate-General for Health at the national level and of the Regional Health Administrations at the regional level. The NHS predominantly provides primary care (primary healthcare (PHC)) and general, specialist, and acute hospital care (hospital healthcare (HHC)). Primary care is provided in family health units and in personalized healthcare units, grouped in primary healthcare centre groups. Secondary and tertiary care is provided in hospital units, which can be geographically grouped into hospital centers. The integration of hospitals and primary health units in the same organization is called a local health unit [21].

Due to the recent adoption of the WHO concept in the NSAHA and the lack of Portuguese studies in this area, there is limited knowledge about the use of the Age-Friendly Principles in the Portuguese NHS and the extent to which primary care directors and hospital administrators are familiar with this concept. Therefore, this study aims to assess the relevance of implementing the Age-Friendly Principles in the NHS in Portugal and to analyze the current adaptation of healthcare organizations to the health profile of older adults.

## 2. Materials and Methods

### 2.1. Research Questions

To assess the relevance of the implementation of the Age-friendly Principles in the Portuguese context and analyze the current adaptation of healthcare organizations to these Principles, considering the WHO Toolkit [14], the following research questions were formulated:

RQ 1: To what extent are primary care directors and hospital administrators familiar with the Age-Friendly Principles developed by the WHO?

RQ 2: To what extent do primary care directors and hospital administrators value the implementation of the Age-friendly Principles in the NHS?

RQ 3: How are the Age-Friendly Principles being implemented across the NHS?

To answer the research questions, a questionnaire survey was developed to obtain the views of NHS healthcare organizations.

### 2.2. Participants

**Figure 2:**
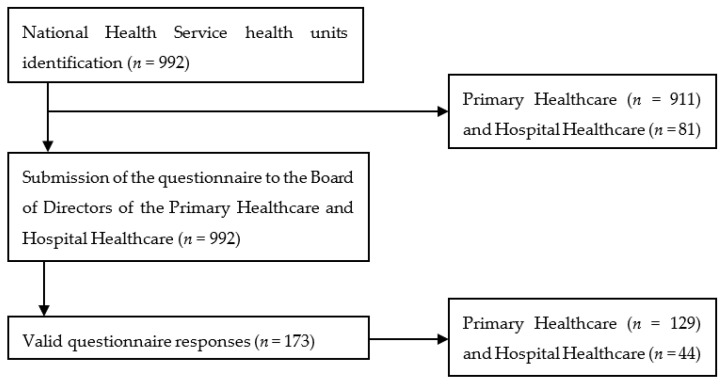
Characterization of the participants.

In 2020, the Portuguese National Health Service (NHS) consisted of 911 primary healthcare units and 81 hospital units. Questionnaires were distributed to all primary healthcare units and hospital units within the NHS, including the Board of Directors of the primary healthcare units and the Hospitals (Figure 2). The selection of this sample was based on up-to-date information obtained from the Ministry of Health website, which was updated regularly throughout the year.

The questionnaire was limited to primary care directors and hospital administrators who exercise functions in the NHS, as this is the central element of the Health System and the focus of the study. This choice of directors and administrators is justified by the functions they perform in the management and organization of healthcare, in conjunction with the health policies defined by the direct administration (e.g., Directorate–General for Health) and indirect administration (e.g., Regional Administration of Health).

### 2.3. Data Collection

Data collection was conducted using an online questionnaire provided and directly administered on the Lime Survey platform [22]. The questionnaire was developed based on the WHO toolkit checklist [14], adapted to the Portuguese context, and supplemented with relevant studies identified through a systematic literature review [16].

Prior to implementation, a reading comprehension test [23] was conducted to assess the suitability of the questions in relation to the study objectives, as well as their clarity and response time. The test was distributed on a small scale to directors of primary care and hospitals within the NHS.

Following its evaluation, the questionnaire was distributed to institutional email addresses obtained from the Ministry of Health website. A brief explanation of the scope and objectives of the study was sent with the questionnaire. The central services of the health organizations received the questionnaire and forwarded it to the Ethics Committee, which reviewed and approved it before disseminating it to the board members. To achieve a higher response rate, monthly reminders were sent to the email addresses of the facilities and quarterly telephone calls were made. In addition, the Portuguese Association of Hospital Administrators, the National Association of Family Health Units, Primary Healthcare Centre Groups, and Regional Health Administrations were approached to disseminate the questionnaire. Data collection began in November 2020 and lasted until September 2021.

### 2.4. Variables

The questionnaire was structured in four sections. The first section collected respondents’ sociodemographic information, e.g., biographical data (sex and age group), healthcare organization where they work, position held, and length of service.

The second section carried out a self-assessment of the respondents’ level of knowledge about the Principles and some related concepts from the literature, identifying the main source of this knowledge. The knowledge of the measures proposed in the NSAHA 2017–2025, the only Portuguese document that reflects the Age-Friendly Principles, was also questioned. All questions had a dichotomous measurement scale, encoded with 0 (No) and 1 (Yes).

Given that more specific aspects should only be asked at a later stage of a questionnaire [24], only in the third and fourth sections were respondents asked questions related to the relevance of the application of these Principles and their current use in the national context, respectively.

In the third section, it was questioned, initially, if the respondents consider that the current structure of healthcare represents a problem for older adults. Then, the level of adequacy of information, communication, and training of professionals regarding older people was questioned, as well as the adjustment of the care management system to the needs of the older people and the accessibility of the internal and external physical environments of the health organizations. These questions presented a dichotomous measurement scale, coded with 0 (No) and 1 (Yes). It should be noted that in the first question, formulated in a negative sense, the punctuation was inverted. At the end of this section, the impact of adopting the three Age-Friendly Principles in the provision of healthcare in Portugal was evaluated, using a 5-point Likert scale (1—very little, 2—little, 3—more or less, 4—very, 5—quite impactful).

In the fourth section, we began by assessing the general implementation of the WHO Principles in health organizations, namely, the development of an age-friendly policy, the prioritization of compatibility with older people in strategic plans, and the definition of a team of coordination for the implementation of the WHO Principles. In turn, the remaining section was divided by the three WHO Principles, and for each Principle the respective checklist developed by the WHO was presented: the Information, Communication, and Training Principle includes 11 criteria on training staff in clinical management and approaches to patient and family education; the Healthcare Management System Principle includes 25 criteria addressing adapting procedures to the special needs of older people; and the Physical Environment Principle also includes 25 criteria and advocates for the application of universal design principles. All questions in this section used a multiple response scale, where the options No (0), Yes (1) and I do not know (2) were considered. As in the previous section, in the negative sense criteria, the coding of 0 and 1 was inverted.

The questionnaire was preceded by a brief introductory note, which included the request for collaboration in its completion, the justification for applying the instrument, the institution where the research was conducted, and the declaration of confidentiality and anonymity [25]. All answers were mandatory to avoid non-completion by distraction. The questionnaire is provided in Appendix A (Table A1).

### 2.5. Data Analysis

Data were analyzed using the Statistical Package for Social Sciences (SPSS) software, version 27.0. Statistical analysis was based on two main methodologies: descriptive and inferential. Descriptive analysis (frequencies, percentages, mean, and standard deviation) was conducted to characterize the sample in relation to the variables under study and inferential analysis (chi-square test, Mann–Whitney test, Kruskal–Wallis test, and Fisher/Fisher–Freeman–Halton exact test) to study the possible associations between variables. Considering that the sample under study does not have a normal distribution and is asymmetric, we applied non-parametric tests, which are more robust for the analysis of associations between qualitative data (nominal and ordinal). Thus, the chi-square test (*x*^2^) was used to analyze nominal qualitative variables in tables 2 × 2 or larger. When the conditions for applying the chi-square test were not met—that is, more than 20% of the cells had an expected frequency of less than five—the Fisher’s exact test (F) was used, which provides more exact *p*-values. Since Fisher’s exact test is used in 2 × 2 tables, the Fisher–Freeman–Halton (F) test was used to analyze larger tables. The Mann–Whitney (U) test was used to study ordinal qualitative variables for two samples, and for three or more samples, the Kruskal–Wallis (H) test was applied. It should also be mentioned that a significance two-tailed level of 5% was used as a reference, i.e., the data were statistically significant at *p* < 0.05.

### 2.6. Ethical Considerations

All subjects gave their informed consent for inclusion before they participated in the study. The study was conducted in accordance with the Declaration of Helsinki of 1975 and the Portuguese rules of the General Law for the Protection of Personal Data, 8th August 2019. The protocol was approved by the Ethics Committee of the Hospital Units, Primary Healthcare Centre groups, and Regional Health Administrations within 8 months (4828/CES/2021).

## 3. Results

### 3.1. Sociodemographic Characterization

Of the total 173 valid answers, 71.1% (*n* = 123) of respondents were female and 28.9% (*n* = 50) were male, with a prevalence in the age group below 40 years (37, 6%, *n* = 65).

Regarding the organization, 74.6% (*n* = 129) of respondents work in PHC and 25.4% (*n* = 44) in HHC. For primary care, a response of 14.2% (*n* = 129) was achieved from the total sample (*n* = 911), and for hospital care, a response of 54.3% (*n* = 44) was obtained of the total sample (*n* = 81).

In this context, 37% (*n* = 64) of respondents were PHC coordinators and 8.7% (*n* = 15) were HHC director nurses. The position of president of the Hospital Administration Council was the least represented in the questionnaire (2.9%, *n* = 5).

A total of 48% (*n* = 83) of the respondents had held a management position for less than 3 years, while 11.6% (*n* = 20) had remained in that position for more than 13 years. However, in the hospital context, no respondent had worked for more than 12 years.

The summary characterization of the sample is shown in Appendix B (Table A2).

### 3.2. Knowledge of Age-Friendly Principles

The Age-Friendly Principles were not recognized by a large majority of respondents (71.1%, *n* = 123). Of the 28.9% (*n* = 50) who claim to know about these Principles, 60% (*n* = 30) did so through WHO guidelines (Table 1). It is possible to observe statistically significant differences (*x*^2^ = 15,685; *p* < 0.001) between respondents who worked in primary and hospital care. Most respondents in hospital care (52.3%, *n* = 23) claimed to know about the Principles, while in primary care, the vast majority (79.1%, *n* = 102) denied knowledge. Furthermore, regarding the job role of the respondents (F = 41.125; *p* < 0.001), the nurse directors of hospital care were those who are more familiar with the Principles (86.7%, *n* = 13), as opposed to clinical directors of hospital care (0%, *n* = 0) or members of the primary care technical council (14.3%, *n* = 8).

Together with the Age-Friendly Principles, an attempt was made to understand the knowledge of other concepts derived from the literature review. Most respondents did not know any of the suggested concepts (65.3%, *n* = 113). However, 25.4% (*n* = 44) of respondents claimed to be familiar with the “Age-Friendly Hospital” project, 20.2% (*n* = 35) with the “Age-Friendly Primary Healthcare”, 11% (*n* = 19) with the “Age-Friendly Health System”, 4% (*n* = 7) with the “Age-Friendly Emergency Service”, 2.3% (n = 4) with the “Age-Friendly Surgery Service”, and 1.7% (*n* = 3) with “Age-Friendly Acute Care” (Appendix B, Table A3).

When the WHO Principles were explained, the percentage of respondents acknowledging the Principles increased (46.8%, *n* = 81) (Appendix B, Table A4). There were statistically significant differences (*x*^2^ = 39.042; *p* < 0.001) in knowledge of the Principles after their explanation. Thus, 48.1% (*n* = 39) of respondents who previously denied knowing about the Principles then answered in the affirmative. Most respondents in primary care (58.9%, *n* = 76) did not know about the WHO guidelines, while in hospital care, 63.6% (*n* = 28) claimed to know about the guidelines (*x*^2^ = 6.701; *p* = 0.010). As acknowledged previously, the position of nurse director (80%, *n* = 12) had greater knowledge of the Principles (F = 13.648; *p* = 0.028) in contrast to that of the clinical director (36, 4%, *n* = 4) of hospital care. Within the scope of primary care, it was again the position of coordinator (42.2%, *n* = 27) which had the greatest knowledge of the Principles.

Finally, with the objective of understanding the respondents’ familiarity with the measures proposed in the NSAHA, which oversees the adaptation of health services to improve the care, participation, independence, and dignity of older adults, most respondents recognized the measures (60.7%, *n* = 105) (Appendix B, Table A4). The data also allowed us to state that most respondents in hospital care (81.8%, *n* = 36) recognize the measures proposed in NSAHA (*x*^2^ = 11.038; *p* < 0.001), while at the level of primary care, only 53.5% (*n* = 69) of respondents recognize these measures.

In this group of questions, an attempt was also made to establish associations with sex, age group and time of experience in management positions of the respondents, however, no statistically significant results were found.

### 3.3. Importance of the Age-Friendly Principles

The importance of the Age-Friendly Principles was assessed, taking into account the respondents’ opinions on the suitability of the current structure of health organizations to provide care for older adults and the impact of adopting improvement measures within the scope of the three WHO Principles regarding care for older adults.

#### 3.3.1. Adequacy of the Structure of Health Services for Older Adults

For 79.8% (*n* = 138) of respondents, the current organizational structure of the health services did not present a problem in providing care to older adults (Table 2).

When questioned about the adequacy of information, education, communication, and training of professionals regarding older adults, 64.2% (*n* = 111) of the respondents answered that it was adequate. With regard to the management system and care process, 50.3% (*n* = 87) of respondents considered that it adjusted to the needs of older adults. Regarding the physical environment, 79.8% (*n* = 138) of respondents considered the internal physical space accessible to older adults, and 63.6% (*n* = 110) felt that the surrounding space allows unrestricted mobility for older adults (Table 2).

There were statistically significant differences in the adequacy of the training of professionals (*x*^2^ = 5.147; *p* = 0.023) and the care management system (*x*^2^ = 4.577; *p* = 0.032) according to the health organization. Thus, regarding the training of professionals, 69% (*n* = 89) of respondents in primary care considered it adequate, while in hospital care, only 50% (*n* = 22) held the same opinion. Regarding the management system and care process, 55% (*n* = 71) of respondents in primary care felt that it has been adjusted to the needs of the older adults, as opposed to 63.6% (*n* = 28) of care hospitals who reported that it does not respond adequately to the needs of the elderly.

#### 3.3.2. Impact of Implementing the WHO Principles

A total of 44.5% (*n* = 77) of the respondents considered the adoption of information, education, communication, and training measures for professionals in areas such as clinical geriatrics, communication with older adults, and healthy ageing to be of great impact. As for changes in the care management system, such as priority in care for older adults, specific opening hours (i.e., specific consulting times in a day or week dedicated to the older persons’ needs), and geriatric screening (i.e., triage protocols in the emergency department and in the ambulance that consider the age criterion), 29.5% (*n* = 51) of respondents felt it was neither positive nor negative and 29.5% (*n* = 51) felt it was very impactful. In turn, adapting the physical environment through the application of universal design principles (i.e., the design and composition of an environment so that it can be accessed, understood, and used to the greatest extent possible by all people regardless of their age, size, ability, or disability), and easy access to public transport was assessed as having a high impact by 41% (*n* = 71) of respondents (Table 3).

The calculation of the mean and standard deviation showed that the adoption of information, education, communication, and training measures for professionals had the strongest impact (M = 4.25; SD = 0.843), followed by adaptation of the internal and external physical environment (M = 3.98; SD = 1.089), and, with less impact, changes in the care management system (M = 3.69; SD = 1.096). The Wilcoxon test also confirmed that the impact values were significantly (*p* < 0.001) above the midpoint of the scale (“more or less”), with most respondents considering the three factors as “very” or “quite” impactful.

The impact of changes in the care management system showed statistically significant differences depending on the health of the respondents (U = 2071,000; *p* = 0.005), with a higher percentage of primary care respondents choosing “more or less” of an impact (31%, *n* = 40) and hospital care choosing “quite” an impact (38.6%, *n* = 17). In hospital care, no respondent considered changes in the care management system to be of “very little” or “little” impact, contrary to what was observed in primary care.

In the care management system (H = 21.418; *p* = 0.002), while most of the presidents of the Board of Directors (60%, *n* = 3) considered that the changes would have “more or less” of an impact, the executive members (55.6%, *n* = 5) and the other management positions not broken down (69.2%, *n* = 9) felt there would be a more optimistic impact (“quite”).

With regard to the impact of adaptations in the physical environment (H = 18.187; *p* = 0.006), most of the presidents of the Board of Directors (80%, *n* = 4) considered that the adaptations would have a “very” large impact, as did director nurses (53.3%, *n* = 8). Of the opinion that the adaptations would have a “great” impact were most of the members of the technical council (50%, *n* = 28), the clinical directors (54.5%, *n* = 6), the executive members (66.7%, *n* = 6), and other management positions not broken down (76.9%, *n* = 10).

In this section, no statistically significant differences were found with regard to sex, age group, and time of experience in management positions of the respondents.

### 3.4. Implementation of the Age-Friendly Principles

The implementation of the Age-Friendly Principles was analyzed based on the opinion of the respondents of the verification of the criteria defined in the WHO Toolkit [14] for each of the three Principles, preceded by a general analysis of the application of the concept in the strategic plans of the health organizations under study.

#### 3.4.1. General Concept

Most respondents (57.8%, *n* = 100) indicated that the health organization where they work had developed a friendly policy for older adults. On the other hand, 43.4% (*n* = 75) of the participants said that the strategic plan of their health organization did not identify compatibility with older adults as a priority, and 70.5% (*n* = 122) denied the existence of a team for the coordination and implementation of the Age-Friendly Principles (Appendix B, Table A5).

#### 3.4.2. Principle of “Information, Education, Communication, and Training”

In this section, the results highlight the lack of specific protocols for the evaluation and clinical management of older adults (56.1%, *n* = 97), the lack of training of health professionals (74.6%, *n* = 129) and other employees (75.7%, *n* = 131) in verbal and non-verbal communication, and the lack of training of health professionals on the four main geriatric syndromes (52%, *n* = 90) (Table 4).

In this domain, there were statistically significant differences in the five criteria according to the health organization. The first concerns the protocols to deal with the abuse of older adults (*x*^2^ = 14.864; *p* < 0.001), where 68.2% (*n* = 30) of hospital care organizations included this and 51.2% (*n* = 66) of primary care did not.

The second criterion is related to the health professionals’ awareness of the normal ageing process and its characteristics (*x*^2^ = 12.325; *p* = 0.002). In hospital care, 81.8% (*n* = 36) of respondents stated that their health organization is aware of this, while in primary care, this was only present in only 52.7% (*n* = 68).

The third criterion is the training of health professionals in the two most prevalent chronic diseases in older adults (F = 7.441; *p* = 0.020). At the level of hospital care, only 2.3% (*n* = 1) reported not having such training while in primary care the figure rises to 13.2% (*n* = 17).

The last criterion concerns the knowledge of health professionals about municipal programs and health policies for older adults (*x*^2^ = 7.061; *p* = 0.029), where most respondents in hospital care (56.8%, *n* = 25) did not know how to answer this question, as opposed to only 34.1% (*n* = 44) of primary care respondents.

A total of 88.7% (*n* = 55) of respondents who considered that the information, education, communication, and training of professionals (health professionals and other employees) was not adequate denied the existence of training given by the organization itself in verbal and non-verbal communication with older adults (*x*^2^ = 11.686; *p* = 0.003). It should be noted that this is the only criterion where statistically significant differences were observed to the question in the previous section related to this Principle.

#### 3.4.3. Principle of “Healthcare Management System”

Contrary to what was observed in the previous Principle, here, it is evident that the vast majority (72%) of the defined criteria are not being implemented by the health units. There is a significant absence of a priority system for older adults at assistance desks (72.3%, *n* = 125) and during appointment scheduling (86.7%, *n* = 150), of an exclusive queue at assistance desks (90.2%, *n* = 156), a care coordinator (note that the care coordinator is responsible for planning medical appointments and referrals, for exchanging information between professionals, and for developing therapeutic plans suited to patients’ needs) (77.5%, *n* = 134), specific screening protocols (90.8%, *n* = 157) or a specialized clinical area (87.9%, *n* = 152), an appointment reminder system (86.1, *n* = 149), an exclusive nursing room (97.1%, *n* = 168), a dedicated recreation area (94.2%, *n* = 163), specific opening hours (96.5%, *n* = 167), extra time for medical appointments (88.4%, *n* = 153), advance disclosure of service costs (85.5%, *n* = 148), and availability of an ethics specialist to advise professionals and users (76.9%, *n* = 133). Furthermore, there was a lack of a prayer room for each department (63.6%, *n* = 28), reduced waiting time between arrival and medical appointment (65.3%, *n* = 113), and a cost reduction system (66.5%, *n* = 115) (Table 5).

There were statistically significant differences in eleven of the criteria presented according to the health organization.

The first criterion concerns the implementation of the assessment of the annual state of health of people aged over 65 years (*x*^2^ = 44.809; *p* < 0.001), where 84.5% (*n* = 109) of primary care workers claimed to do this evaluation, compared to 50% (*n* = 22) of hospital care workers.

The second criterion is based on the existence of an exclusive queue for older adults at the assistance desk (*x*^2^ = 13.021; *p* = 0.001), where 22.7% (*n* = 10) of hospital care workers said they supplied this and 94.6% (n = 122) of primary care said they did not.

The third criterion refers to the provision of clearly explained medical guidelines (*x*^2^ = 11.845; *p* < 0.001), in which all hospital care respondents referred to the delivery of these guidelines (100%, *n* = 28), as opposed to 68.4% (*n* = 78) of primary care workers.

The fourth criterion concerns the availability of home care (i.e., home care is available in primary care for medical appointments or nursing care; in hospital care, home hospitalization is foreseen, which provides for the provision of differentiated and complex care at a hospital level at home) (*x*^2^ = 41.971; *p* < 0.001), where most primary care organizations provide this service (97.7%, *n* = 125), in contrast to 61.4% (*n* = 27) of hospitals.

The fifth criterion addresses the specific triage protocols (i.e., a clinical risk management tool used by clinicians to enable them to safely manage patient flow when clinical need far exceeds capacity (e.g., Manchester Triage System)) for older adults (F = 6.956; *p* = 0.008), in which 15.9% (*n* = 7) of the respondents of hospital care affirmed the existence of these protocols, while only 4% (*n* = 5) of primary care organizations affirmed this.

The sixth criterion, regarding the availability of a list of (in)formal caregivers (*x*^2^ = 7.191; *p* = 0.007), confirms that in 64.1% (*n* = 25) of hospital care organizations, this list is available; however, in primary care, this is only observed in 39% (*n* = 41).

The seventh criterion refers to the monitoring of older adults by a family member or caregiver during the provision of care (F = 16.663; *p* < 0.001), in which 96.9% (*n* = 123) of primary care organizations allow this monitoring, while in hospitals, the same happens in 77.3% (*n* = 34).

The eighth criterion is the minimization of the waiting time (*x*^2^ = 5.755; *p* = 0.016), in which it is noted that 83.3% (*n* = 35) of hospitals do not implement this, compared to 63.4% (*n* = 78) of primary care organizations.

The ninth criterion concerns extra time for appointments for older adults (F = 4.392; *p* = 0.036), and in all hospital care organizations (*n* = 42), this criterion is not verified; however, in 9.8% (*n* = 12) of primary care organizations, extra time is implemented.

The tenth criterion refers to the disclosure of the amount to be paid for the previous day’s medical appointments (F = 4.353; *p* = 0.037), where it is observed that no hospital organization (*n* = 43) carries out this disclosure, while 9.5% (*n* = 11) of primary care organizations do.

The last criterion relates to the availability of an ethics specialist who can advise professionals and users on issues related to healthcare provided to older adults (*x*^2^ = 20.024; *p* < 0.00). A total of 90.7% (*n* = 107) of primary care organizations do not have an ethics specialist, while in 39.5% (*n* = 17) of hospital care providers there is one available.

Statistically significant differences were observed in the five criteria according to the previous evaluation of the respondents on the adjustment of the management system and care process to the health needs of older adults.

Thus, regarding clearly explained prescribed medications (F = 9.866; *p* = 0.004), it is noted that 45.9% (*n* = 72) of respondents who consider the management system and care process inadequate confirm the application of this criterion.

As for the existence of a specialized clinical area for older adults (*x*^2^ = 8.524; *p* = 0.003), the respondents who considered the management system and care process not to have been adjusted to the needs of older adults divided their answers (50%, *n* = 76) between the existence of a specialized clinical area and the lack of it.

For the availability of volunteers who guide older adults in healthcare (F = 6.465; *p* = 0.016), 67.9% (*n* = 19), who do not appreciate the management system, declared the existence of these volunteers.

Regarding the list of (in)formal caregivers or social responses (*x*^2^ = 6.517; *p* = 0.011), 34.8% (*n* = 24) of respondents who stated there was a mismatch of the management system confirmed the existence of this list.

The fifth and last criterion concerns the minimization of the waiting time between arriving at the location and carrying out the medical appointment (*x*^2^ = 4.339; *p* = 0.037), in which it is observed that 76.3% (n = 61) of the respondents who assessed the management system as inadequate also disagree with the implementation of this criterion.

#### 3.4.4. Principle of “Physical Environment”

In the analysis of the last WHO Principle, most of the criteria have been implemented in the health units (64%). However, the results showed a lack of exclusive parking spaces for older adults (86%, *n* = 147), non-slip floors in all areas (51.5%, *n* = 88), doors opening in both directions (82.4%, *n* = 140), supervision of bathroom use (62.4%, *n* = 106), availability of support technologies (64.2%, *n* = 106) and free telephones (73.2%, *n* = 123), free water points (63.2%, *n* = 108), and snacks available (69.1%, *n* = 114) (Table 6).

From the checklist presented in this Principle, there were statistically significant differences in four criteria according to the health organization in which the respondents perform their duties.

Regarding the supervision of older adults in the bathroom (*x*^2^ = 27.046; *p* < 0.001), there was a tendency for respondents from hospital care to confirm this follow-up (59.1%, *n* = 26), as opposed to primary care, where only 19% (*n* = 24) claim to have implemented this criterion.

Regarding the availability of support technologies for older adults (*x*^2^ = 26.426; *p* < 0.001), most respondents in hospital care (56.8%, *n* = 25) stated that these are available, while in primary care, only 16.5% (*n* = 20) of respondents said they are available.

Hospital care stood out, again, regarding the existence of mobile phones or telephones for use by older adults (*x*^2^ = 15.178; *p* < 0.001), with 40.9% (*n* = 18) of hospital care respondents responding affirmatively, compared to 13.7% (*n* = 17) of primary care respondents.

The last criterion concerns the availability of snacks for older adults (F = 33.498; *p* < 0.001), in which 81.8% (*n* = 99) of primary care providers said they did not provide these, and 61.4% (*n* = 27) of hospital care providers claiming to provide these snacks.

Finally, there were significant associations between the initial opinion of respondents about the accessibility of the internal and external physical spaces of the health organizations, and eleven of the criteria analyzed this dimension.

In the first criterion, signposts placed in all important areas (F = 8.525; *p* = 0.015), only 2.9% (*n* = 4) of respondents who considered the indoor physical environment accessible disagreed with the application of this criterion.

In the second criterion (elevators and wide and spacious corridors), 6.5% (*n* = 8) of respondents considered the internal physical environment accessible (F = 11.375; *p* = 0.002) but disagreed that the elevators and corridors were adequately sized.

In the third criterion, access to different areas of the unit and easy navigation (F = 25.098; *p* < 0.001), 51.4% (*n* = 18) of respondents who disagreed with the adequacy of the internal physical space also said there was no easy access to different areas inside the units.

In the fourth criterion, non-slip floors in all areas (*x*^2^ = 6.548; *p* = 0.038), 46.7% (*n* = 64) of respondents who considered the indoor environment accessible did not confirm the application of this criterion.

In the fifth criterion, only 10.2% (*n* = 14) of respondents who agreed with the accessibility of the interior of the unit said that the bathroom floor was not kept clean and dry (F = 6.934; *p* = 0.028).

In the sixth criterion, availability of support technologies for older adults (*x*^2^ = 6.801; *p* = 0.033), 59.2% (*n* = 77) of respondents considered the indoor environment accessible but denied the implementation of this criterion.

In addition, for the seventh criterion (size and shape of the letters on the signs), 14.5% (*n* = 20) of respondents who considered the internal physical space accessible (F = 15.190; *p* < 0.001) did not consider the signs appropriate signage. On the other hand, 10% (*n* = 11) of the respondents who revealed the adjustment of the external physical environment (F = 21.983; *p* < 0.001) denied the adequacy of the signs.

In the eighth criterion, entrance adapted to older adults, 80% (*n* = 28) of the respondents who denied the accessibility of the internal physical space (F = 7.145; *p* = 0.016) claimed they met this criterion. Furthermore, with regard to unrestricted mobility in the surrounding space, 83.9% (*n* = 52) of respondents who did not agree with this considered that their entrances were adapted for older adults.

In the ninth criterion, good lighting inside and outside spaces, only 3.6% (*n* = 5) of respondents claimed that their indoor physical environment was accessible (F = 7.319; *p* = 0.019) but denied the implementation of this criterion. It was observed that 85.7% (*n* = 54) of respondents who denied the adequacy of the external environment (F = 10.412; *p* = 0.001) confirmed the good lighting of spaces.

In the tenth criterion, the width of the doors, 65.7% (*n* = 23) of the respondents denied the accessibility of the internal physical space (F = 20.018; *p* < 0.001) but affirmed the implementation of this criterion. Accordingly, 74.6% (*n* = 47) of respondents denied easy mobility in the surrounding spaces (F = 19.365; *p* < 0.001) but considered the width of the doors adequate.

In the last criterion, parking spaces for older adults, 80.6% (*n* = 87) of respondents considered the mobility of the surrounding space unrestricted (F = 7.248; *p* = 0.026); however, they disagreed with the application of this criterion.

## 4. Discussion

The results show that most healthcare administrators and directors do not recognize the Age-Friendly Principles. The (scarce) familiarity that is shown comes largely from the WHO guidelines. The directors of primary care have little awareness, especially the members of the technical council and coordinators. The concept of an “Age-Friendly Hospital” is the most recognized by respondents, albeit scarcely. However, it is shown that discriminating between the WHO Principles results in increased recognition by respondents. Hospital care continues to stand out positively compared to primary care, and nurse directors, executive members, and other management positions have a greater recognition of the WHO Principles. The reduced familiarity shown here can be justified by the scarce scientific research in this area, as well as the use of different principles from those defined by the WHO, as evidenced in the study by Tavares et al. [16]. Likewise, greater recognition of the “Age-Friendly Hospital” concept—in particular, by hospital administrators—may be because European scientific production focuses on this domain, as the same study concluded.

Most respondents did not identify the current organizational structure of care as a problem for the provision of care to older adults. In this sense, most consider the information, education, communication, and training of professionals adequate, although half of hospital directors do not share this opinion. With a less positive assessment come the management system and care processes, in which half of the respondents state that these are adjusted to the needs of older adults; however, most hospital directors disagree that there has been an adjustment. A better evaluation is the internal physical space, where a large percentage of respondents stated that their organizations have accessible spaces for older adults. However, most respondents felt that the surrounding physical space did not promote the mobility of older people. With respect to the adoption of improvement measures in the three WHO Principles, in terms of information, education, communication, and training of professionals and the physical environment, they are considered to have a great impact. Regarding the management system, the opinion is shared between an impact which is neither positive nor negative and a lot of impact. In line with the results achieved, the studies by Woo et al. [26] and Kuo and Chen [27] point out flaws in the current organization of primary and hospital healthcare organizations, respectively, highlighting care management systems with less satisfactory results, given the better panorama presented in the physical environment of the health units. There is also a need to adopt measures to improve the training of professionals in the area of geriatrics and gerontology according to the study by Ahmadi et al. [28].

The results regarding the implementation of the general concept and the three Age-Friendly Principles developed by the WHO showed that regarding the general concept, most respondents believe that their health organization implements age-friendly policies. However, a significant number of respondents stated that their strategies do not prioritize compatibility with older adults. Additionally, most leaders stated that they do not have a team for coordinating and implementing the Age-Friendly Principles. Similarly, studies conducted by Ssensamba et al. [29] and Wong et al. [10] confirm the commitment of healthcare organizations to this concept but highlight the absence of a geriatric care policy, national guidelines for geriatric care management, a coordinating team for implementing such care, and a support team for its implementation. Furthermore, the same studies reveal that older adults are not represented on the committees of healthcare units, limiting the development of measures that truly respond to their needs.

Next, regarding the Principle of “Information, Education, Communication, and Training,” it is noted that only half of the criteria are being implemented. In this domain, hospital care managers tend to evaluate the criteria more positively. However, it is noteworthy that most respondents who consider the information, education, communication, and training of professionals adequate deny that their healthcare organization provides training to its professionals in verbal and non-verbal communication with older adults. A recent study by Geng et al. [30] highlights the need to invest more in the professional training of doctors, nurses, and social workers, increase their clinical experience with older adults, improve their awareness during clinical practice in psychological skills and communication, and strengthen the sympathy of professionals. Likewise, a study conducted by Weldingh and Kirkevold [31], considering the reports of older adults and their families, reveals the importance of professionals establishing holistic and individualized communication adapted to the older adult patient, respecting the patient, and not focusing solely on the task or diagnosis.

In turn, the principle of the “healthcare management system” has a reduced number of criteria to be implemented. Here, primary and hospital care leaders share the same opinion, predominantly negative; however, there are disparities in two respects. Most hospital leaders identified that a list of (in)formal caregivers or social responses for older adults is not available in their health organization, while most primary care providers mentioned the opposite. Furthermore, half of all hospital directors state that users over 65 years of age are not counselled, examined, or monitored annually, while most primary care workers say that they are. When comparing the opinion of the respondents on the suitability of the healthcare management system for older adults with the checklist in this area, it appears that although there is an initial negative opinion, the respondents assess these criteria more positively. These flaws are shared in a study by Boltz et al. [32], which adds to the lack of evidence-based protocols, inadequate support for decision-making, long waits for care, lack of volunteers, non-prioritization of prevention of geriatric syndromes, and inadequate hospitalizations. The adoption of care management programs is thus urgent as health systems seek to improve the coordination and integration of services [33].

Finally, the “physical environment” is the Principle that has the most criteria met. It is at the level of hospital care that the largest set of implemented criteria is observed. The positive opinion of hospital directors differs significantly from primary care providers regarding the surveillance and monitoring of older adults in the bathroom, the availability of support technologies for older adults, and the availability of snacks for older adults. If we consider the initial opinion of the respondents about the accessibility of the internal physical space, a positive viewpoint remains after scrutiny of the criteria, excluding two criteria with negative assessments: the existence of slippery floors in all areas; and the availability of assistive technologies for older adults. Furthermore, regarding mobility in the surrounding physical environment, the initial positive view remains in the criteria checklist, except for exclusive parking spaces for older adults, where the vast majority emphasize their nonexistence. A study developed by Tavares et al. [34], which analyzes the physical accessibility of a hospital located in the central region of Portugal, contradicts the positive results achieved here. The study concluded that more than half of the evaluated criteria were not being implemented, suggesting that the design and support products were scarce in view of the needs, complexity, and specificity of hospitalized older adults. However, the heterogeneity of scores may be a result of existing resources, the management of spaces, and the focus given to physical activity and promoting the independence of the older adults in each health unit.

Despite the accomplishments thus far, the NSAHA in Portugal envisions the implementation of Age-Friendly Principles within the NHS. This shows that the political agenda in the country’s healthcare sector is in line with the guidelines established by the WHO and implemented in several countries worldwide. Consequently, decision-makers have the opportunity to learn from the experiences of other nations that have successfully applied these principles, benefiting from their outcomes while also identifying areas for improvement in their own context [35].

However, since this policy has yet to be implemented, its dissemination is expected to occur through a process of learning and emulation [36]. Globalization plays played a pivotal role in facilitating the sharing of experiences, information, and best practices, thereby creating various opportunities and platforms for policy exchange [37]. It is anticipated that the experiences of other countries will enlighten policy-makers in Portugal as to the advantages of adopting these principles within the NHS, thus streamlining the decision-making process. Similar to the adoption of the WHO Baby-Friendly Hospitals Initiative in Portugal [38], it is expected that the Portuguese government will establish a national commission to oversee the application of these principles, assuming responsibility for project monitoring and evaluation, as well as the accreditation of age-friendly organizations.

### Limitations

It should be noted that this analysis has limitations regarding data collection, processing, and analysis. First, the Ethics Committees did not allow the collection of information that could identify, in any way, the health organization or the respondent, so the possibilities of analysis were, from the outset, conditioned. For example, it was not possible to infer possible regional disparities, legal regimes, or structural models. Second, the data do not allow for analyzing differences between and within departments, which may arise as a result of investments and improvements in specific services. Third, the questionnaire survey was not validated; it was derived from the WHO toolkit checklist. Fourth, the method adopted for the application of the questionnaire survey, i.e., it being self-administered, may result in subjective interpretations of the criteria dependent on the respondents. Fifthly, there were limitations to the sample obtained, especially in primary healthcare, justified by the application of the survey in the period of the first infections by SARS-CoV-2 and the consequent concentration of professionals in the treatment and disease management. Finally, the design of this quantitative study may not have enabled the collection of sufficiently robust data to comprehensively assess the implementation of the WHO Principles in all their dimensions. Adopting a mixed-methods approach in the future, combining both qualitative and quantitative methods, may provide a more comprehensive analysis.

## 5. Conclusions

Demographic changes have marked the last decades and have disruptively transformed the model of health needs for which health systems were designed. The Age-Friendly Principles developed by the WHO have been designed to reconfigure different health services, making them capable of responding to this new demographic reality.

Our findings indicate that healthcare decision-makers are largely unfamiliar with the concept. Therefore, there is a pressing need to disseminate global guidelines more effectively in the Portuguese context, with a particular emphasis on clarifying and promoting the concept through the Ministry of Health. Furthermore, the study highlights that implementing measures to improve the three WHO Principles can be promising and may have a significant impact on providing specialized and efficient healthcare to older adults with comorbidities. However, the actual implementation of these measures falls short of expectations. Thus, new ways of encouraging and supporting the implementation of the WHO Principles in the NHS are necessary. Additionally, research to assess the effectiveness and impact of implementing the principles across various healthcare levels is vital. This underscores the importance of providing care that is oriented to the current profiles of older adults.

## Figures and Tables

**Table 1:** Knowledge of the Age-Friendly Principles and main source of this knowledge.

		Total
		*n* (%)
Knowledge of Age-Friendly Principles developed for healthcare	No	123 (71.1)
	Yes	50 (28.9)
Main source of this knowledge	WHO	30 (60.0)
	European Commission	5 (10.0)
	Institution itself	3 (6.0)
	Other guidelines	12 (24.0)

**Table 2:** Adequacy of the current structure of health services to provide care to older adults.

	Total	
	*n* (%)	
	No	Yes
The organizational structure of health services represents a problem in provide care to older adults	138 (79.8)	35 (20.2)
Information, education, communication, and training of professionals suited to the profile of the older adults who needs healthcare	62 (35.8)	111 (64.2)
Management system and care process adjusted to the needs of the older adults	86 (49.7)	87 (50.3)
Internal physical space accessible to the older adults	35 (20.2)	138 (79.8)
The surrounding space allows unrestricted mobility for older adults	63 (36.4)	110 (63.6)

**Table 3:** Impact of implementing the three WHO Principles.

	Total	
	*Impact*	*n* (%)
Adoption of information, education, communication, and training measures for professionals in areas such as clinical geriatrics, communication with the older adults, and healthy ageing	Very little	2 (1.2)
	Little	5 (2.9)
	More or less	18 (10.4)
	Very	71 (41.0)
	Quite	77 (44.5)
Changes in the care management system, such as priority in care for older adults, specific opening hours, and geriatric screening	Very little	7 (4.0)
	Little	15 (8.7)
	More or less	51 (29.5)
	Very	51 (29.5)
	Quite	49 (28.3)
Adaptation of the physical environment through the application of universal design principles and easy access to public transport	Very little	6 (3.5)
	Little	11 (6.4)
	More or less	35 (20.2)
	Very	50 (28.9)
	Quite	71 (41.0)

**Table 4:** Implementation of the “Information, Education, Communication, and Training” principle in health organizations.

	Total		
	*n* (%)		
	No	Yes	Do Not Know
Are there instructions and tools needed to provide preventive services—in particular, behavioral counseling on the three main risk factors: smoking, sedentary lifestyle, and unhealthy diet?	28 (16.2)	141 (81.5)	4 (2.3)
Are there specific protocols for the evaluation and clinical management of older adults users?	97 (56.1)	67 (38.7)	9 (5.2)
Are there protocols for dealing with issues of older people abuse in suspected or confirmed cases?	74 (42.8)	80 (46.2)	19 (11.0)
Are health professionals trained, provided by the organization itself, in verbal and non-verbal communications suitable for older adults?	129 (74.6)	35 (20.2)	9 (5.2)
Do the other employees have training, given by the organization itself, in verbal and non-verbal communications suitable for older adults?	131 (75.7)	21 (12.1)	21 (12.1)
Are health professionals sensitized by the organization itself to the normal ageing process and its characteristics?	60 (34.7)	104 (60.1)	9 (5.2)
Are the other employees sensitized by the organization itself to the normal ageing process and its characteristics?	77 (44.5)	74 (42.8)	22 (12.7)
Are health professionals trained by the organization itself in the four geriatric syndromes (memory loss, urinary incontinence, depression, and falls)?	90 (52.0)	70 (40.5)	13 (7.5)
Are health professionals trained by the organization in the two most prevalent chronic diseases (diabetes and hypertension)?	18 (10.4)	148 (85.5)	7 (4.0)
Are health professionals trained by the organization itself to provide preventive services and advice on the three main risk factors: smoking, sedentary lifestyle, and unhealthy diet?	45 (26)	121 (69.9)	7 (4.0)
Are health professionals aware of existing health programs in the municipality, health policies aimed at the older adults, and their rights?	43 (24.9)	61 (35.3)	69 (39.9)

**Table 5:** Implementation of the Principle “Healthcare Management System” in health organizations.

	Total		
	*n* (%)		
	No	Yes	Do Not Know
Are users over 65 advised, examined, treated, and monitored annually?	35 (20.2)	123 (71.1)	15 (8.7)
Is there a priority system for older people in all services?	125 (72.3)	41 (23.7)	7 (4.0)
Is there an exclusive queue for older adults at the assistance desks?	156 (90.2)	16 (9.2)	1 (0.6)
Are volunteers available to guide older people through different services?	9 (20.5) ^b^	35 (79.5) ^b^	0 (0.0)
Are prescribed medications clearly explained to older adults?	8 (4.6)	157 (90.8)	8 (4.6)
Are all medical guidelines arising from the medical appointment provided to the older adults, family member, or caregiver in writing?	36 (20.8)	106 (61.3)	31 (17.9)
Is home healthcare available, with health professionals traveling to older adults people’s homes, if necessary?	20 (11.6)	152 (87.9)	1 (0.6)
Is there sharing of clinical information on older adults people between different levels of care (e.g., between primary care and hospitals)?	22 (12.7)	142 (82.1)	9 (5.2)
Does the health organization designate a care coordinator for older adults?	134 (77.5)	28 (16.2)	11 (6.4)
Are there specific screening protocols for older people (e.g., geriatric screening)?	157 (90.8)	12 (6.9)	4 (2.3)
Is there a multidisciplinary clinical area specialized in the older adults (e.g., geriatrics)?	152 (87.9)	9 (5.2)	12 (6.9)
Is a list of (in)formal caregivers or social responses available for older people?	78 (45.1)	66 (38.2)	29 (16.8)
Can caregivers or family members monitoring older people throughout the caregiving process?	14 (8.1)	157 (90.8)	2 (1.2)
Is there a specific medical appointment reminder system for older adults?	149 (86.1)	14 (8.1)	10 (5.8)
Is there a system that allows the reduction of healthcare costs for older adults (e.g., exemption of user fees)?	115 (66.5)	24 (13.9)	34 (19.7)
Is there an exclusive nursing room for older adults?	168 (97.1)	3 (1.7)	2 (1.2)
Is there any area and/or form of recreation for older adults?	163 (94.2)	6 (3.5)	4 (2.3)
Is there a prayer room for each department (aggregation of services)?	28 (63.6) ^b^	16 (36.4) ^b^	0 (0.0)
Is the waiting time between arriving at the location and carrying out the medical appointment minimized for older adults?	113 (65.3)	52 (30.1)	8 (4.6)
Is there a specific opening hours for older adults?	167 (96.5)	4 (2.3)	2 (1.2)
Do older adults have extra time for medical appointments?	153 (88.4)	12 (6.9)	8 (4.7)
Is there a priority system for seniors when scheduling appointments?	150 (86.7)	15 (8.7)	8 (4.6)
Is the amount to be paid for care prepared and disclosed to the older adults the day before the medical appointment?	148 (85.5)	11 (6.4)	14 (8.1)
Is an ethics specialist available to advise professionals and users on issues related to the care of older people?	133 (76.9)	28 (16.2)	12 (6.9)
Is there an accessible way for older people to assess their satisfaction in addition to the complaints book?	85 (49.1)	81 (46.8)	7 (4.0)

^b^ Does not consider omitted cases (i.e., “Not applicable”).

**Table 6:** Implementation of the “Physical Environment” Principle in health organizations.

	Total		
	*n* (%)		
	No	Yes	Do Not Know
Are signage posted in all important areas (waiting rooms, doctors’ offices, hallways, bathrooms)?	10 (5.8)	159 (92.4) ^b^	3 (1.7)
Is the font size on signage large and bold for better visibility?	36 (20.9) ^b^	132 (76.7) ^b^	4 (2.3)
Are all words and signs written in Portuguese?	1 (0.6)	167 (97.7) ^b^	3 (1.8) ^b^
Are professionals easily identifiable through nameplates or clothing?	32 (18.7) ^b^	137 (80.1) ^b^	2 (1.2)
Are there parking spaces designated exclusively for older adults?	147 (8.0) ^b^	19 (11.1) ^b^	5 (2.9)
Is there a bus and/or train station close to the health organization?	36 (21.2) ^b^	131(77.1) ^b^	3 (1.8)
Is there an entrance adapted for older adults (e.g., level floor, ramp, handrail)?	14 (8.2) ^b^	155 (90.6) ^b^	2 (1.2)
Is there good lighting inside and outside the spaces (e.g., natural light, artificial white light)?	11 (6.4)	160 (92.5)	2 (1.2)
Are all doors wide (entrance door: ≥0.90 m; interior doors: ≥0.80 m)?	16 (9.2)	148 (85.5)	9 (5.2)
Are there elevators available for older adults on all floors?	16 (12.5) ^b^	108 (84.4) ^b^	4 (3.1) ^b^
Are elevators and hallways wide and spacious for older people to move around easily (elevator: 1.10 m wide × 1.40 m deep; hallway: ≥1.20 m wide)?	17 (11) ^b^	134 (86.5) ^b^	5 (2.6) ^b^
Is the interior of the health organization not complex and is access to different areas easy (e.g., demarcation signs, identification signs)?	33 (19.1)	138 (79.8)	2 (1.2)
Is there a sufficient number of chairs in the waiting room for each service (minimum of 20)?	25 (15.1) ^b^	138 (83.1) ^b^	2 (1.8) ^b^
Are floors non-slip in all areas (e.g., offices, hallways, bathrooms)?	88 (51.5) ^b^	68 (39.8) ^b^	15 (8.8) ^b^
Are there grab bars on the stairs on all floors?	41 (29.9) ^b^	92 (67.2) ^b^	4 (2.9) ^b^
Are bathrooms available in all important areas and on all floors?	16 (9.5) ^b^	150 (89.3) ^b^	2 (1.2)
Are bathroom floors always clean and dry?	24 (14.0) ^b^	134 (77.9) ^b^	14 (8.1)
Are there grab bars in all bathrooms?	78 (45.9) ^b^	76 (44.7) ^b^	16 (9.4) ^b^
Is there supervision of older adults when they use the bathroom?	106 (62.4) ^b^	50 (29.4) ^b^	14 (8.2) ^b^
Do the bathrooms have doors that open both ways?	140 (82.4) ^b^	20 (11.8) ^b^	10 (5.9) ^b^
Are assistive technologies available for older people (e.g., walking aids, food, hygiene, communication)?	106 (64.2) ^b^	45 (27.3) ^b^	14 (8.5) ^b^
Is there a telephone or mobile phone available for older adults to use?	123 (73.2) ^b^	35 (20.8) ^b^	10 (6.0) ^b^
Are there free water points for older adults?	108 (63.2) ^b^	55 (32.2) ^b^	8 (4.7) ^b^
Are snacks available for older adults?	114 (69.1) ^b^	46 (27.9) ^b^	5 (3.0) ^b^

^b^ Does not consider omitted cases (i.e., “Not applicable”).

## Data Availability

The data used to support the findings of this study will be available from the corresponding author upon reasonable request.

## Appendix A

**Table A1:** Identification of variables and questionnaire response scale.

Variable	Response Scale
**Sociodemographic data**	
Sex	Male Female
Age group (in years)	<40 41 to 50 51 to 60 >60
Health organization where you carry out your professional activity	Primary healthcare Hospital healthcare
Management position currently held	Coordinator Technical Council President Clinical Director Nurse Director Executive Member Other
Total experience in the management bodies suggested above (in years)	<3 4 to 6 7 to 9 10 to 12 >13
**Knowledge of the concept**	
Are you aware of the Age-Friendly Principles developed within the scope of healthcare?	No Yes
What is the main source of this knowledge?	WHO European Commission Institutional Other
Are these concepts familiar to you? 1. Age-Friendly Hospital; 2. Age-Friendly Health Center; 3. Age-Friendly Emergency Service; 4. Age-Friendly Surgery Service; 5. Age-Friendly Acute Care; 6. Age-Friendly Health System; 7. None of the above.	No Yes
Do you know the WHO guidelines regarding the reorientation of healthcare for older adults?	No Yes
Are you aware of the measures proposed by the Directorate-General for Health, in the National Strategy for Active and Healthy Ageing 2017–2025?	No Yes
Pertinence of the concept	
Do you consider that the current organizational structure of health services may represent a problem in provide care to older adults with comorbidities?	No Yes
Do you consider the information, education, communication, and training of the professionals in the Unit you manage to be adequate for the profile of the older adults who needs healthcare?	No Yes
Do you consider that, in general, the management system and care process of the Unit you direct is adjusted to the health needs of the older adults?	No Yes
Do you consider that, in general, the internal physical space of the Unit you manage is accessible to the older adults?	No Yes
Do you consider that, in general, the physical space surrounding the Unit you manage allows unrestricted mobility for older adults?	No Yes
Indicate the impact that the following actions would have on the promotion of Healthcare for Older Adults: 1. Adoption of information, education, communication, and training measures for professionals in areas such as clinical geriatrics, communication with older adults, and healthy ageing; 2. Changes in the care management system, such as priority in care for older adults, specific hours of care, and geriatric screening; 3. Adaptation of the physical environment through the application of universal design principles and easy access to public transport.	Very little Little More or less Very Quite
**Implementation of the concept**	
General concept:	
1. Does the health organization develop an age-friendly policy that values and promotes the health, dignity, and participation of older adults?	No Yes Do not know
2. Does the health organization’s strategic plan identify compatibility with the older adults as a priority issue?	No Yes Do not know
3. Does the health organization have a team for the coordination and implementation of the Age-Friendly Principles?	No Yes Do not know
Information, Education, Communication, and Training:	
1. Are there instructions and tools needed to provide preventive services—in particular, behavioral counseling on the three main risk factors: smoking, sedentary lifestyle, and unhealthy diet?	No Yes Do not know
2. Are there specific protocols for the evaluation and clinical management of older adults users?	No Yes Do not know
3. Are there protocols for dealing with issues of older adults abuse in suspected or confirmed cases?	No Yes Do not know
4. Are health professionals trained, provided by the organization itself, on verbal and non-verbal communications suitable for older adults?	No Yes Do not know
5. Do the other employees have training, given by the organization itself, on verbal and non-verbal communications suitable for older adults?	No Yes Do not know
6. Are health professionals sensitized by the organization itself to the normal ageing process and its characteristics?	No Yes Do not know
7. Are other employees made aware by the organization itself of the normal ageing process and its characteristics?	No Yes Do not know
8. Are health professionals trained by the organization in the four geriatric syndromes (memory loss, urinary incontinence, depression, and falls)?	No Yes Do not know
9. Are health professionals trained by the organization in the two most prevalent chronic diseases (diabetes and hypertension)?	No Yes Do not know
10. Are health professionals trained by the organization to provide preventive services and advice on the three main risk factors: smoking, sedentary lifestyle and unhealthy diet?	No Yes Do not know
11. Are health professionals aware of the existing health programs in the municipality, the health policies aimed at the older adults and their rights?	No Yes Do not know
Healthcare Management System:	
1. Are users aged over 65 advised, examined, treated and monitored annually?	No Yes Do not know
2. Is there a priority system for older adults in all services?	No Yes Do not know
3. Is there an exclusive queue for older adults at the service counters?	No Yes Do not know
4. Are volunteers available to guide older adults through different services?	No Yes Do not know
5. Are prescribed medications clearly explained to older adults?	No Yes Do not know
6. Are all medical guidelines arising from the medical appointment provided to the older adults, family member, or caregiver in writing?	No Yes Do not know
7. Is home healthcare available, with health professionals traveling to the homes of older adults, if necessary?	No Yes Do not know
8. Is there sharing of clinical information on older adults between different levels of care?	No Yes Do not know
9. Does the health organization designate a care coordinator for older adults?	No Yes Do not know
10. Are there specific screening protocols for older adults (e.g., geriatric screening)?	No Yes Do not know
11. Is there a multidisciplinary clinical area specialized in the older adults (e.g., geriatrics)?	No Yes Do not know
12. Is a list of (in)formal caregivers or social responses available for older adults?	No Yes Do not know
13. Can caregivers or family members monitoring older adults throughout the caregiving process?	No Yes Do not know
14. Is there a specific medical appointment reminder system for older adults?	No Yes Do not know
15. Is there a system that allows the reduction of healthcare costs for older adults?	No Yes Do not know
16. Is there an exclusive nursing room for older adults?	No Yes Do not know
17. Is there any area and/or form of recreation for older adults?	No Yes Do not know
18. Is there a prayer room for each department (aggregation of services)?	No Yes Do not know
19. Is the waiting time between arriving at the location and carrying out the medical appointment minimized for older adults?	No Yes Do not know
20. Is there a specific opening hours for older adults?	No Yes Do not know
21. Do older adults have extra time for medical appointments?	No Yes Do not know
22. Is there a priority system for older adults when scheduling appointments?	No Yes Do not know
23. Is the amount to be paid for the care prepared and disclosed to the older adults the day before the medical appointment?	No Yes Do not know
24. Is an ethics specialist available to advise professionals and users on issues related to healthcare for older adults?	No Yes Do not know
25. Is there an accessible way for older adults to assess their satisfaction in addition to the complaints book?	No Yes Do not know
Physical Environment:	
1. Are signage posted in all important areas (waiting rooms, doctors’ offices, hallways, bathrooms)?	No Yes Do not know
2. Is the font size on signage large and bold for better visibility?	No Yes Do not know
3. Are all words and signs written in Portuguese?	No Yes Do not know
4. Are professionals easily identifiable through nameplates or clothing?	No Yes Do not know
5. Are there parking spaces reserved exclusively for older adults?	No Yes Do not know
6. Is there a bus and/or train station close to the health organization?	No Yes Do not know
7. Is there an entrance adapted for older adults (e.g., level floor, ramp, handrail)?	No Yes Do not know
8. Is there good lighting inside and outside the spaces (e.g., natural light, artificial white light)?	No Yes Do not know
9. Are all doors wide (entrance door: ≥0.90 m; interior doors: ≥0.80 m)?	No Yes Do not know
10. Are there elevators available for older adults on all floors?	No Yes Do not know
11. Are the elevators and corridors wide and spacious for older adults to move around easily (elevator: 1.10 m wide × 1.40 m deep; corridor: ≥1.20 m wide)?	No Yes Do not know
12. Is the interior of the health organization not complex and is access to different areas easy (e.g., demarcation signs, identification signs)?	No Yes Do not know
13. Is there a sufficient number of chairs in the waiting room for each service (minimum of 20)?	No Yes Do not know
14. Are floors non-slip in all areas (e.g., offices, hallways, bathrooms)?	No Yes Do not know
15. Are there grab bars on the stairs on all floors?	No Yes Do not know
16. Are toilets available in all major areas and on all floors?	No Yes Do not know
17. Is there an emergency alarm in each bathroom?	No Yes Do not know
18. Are bathroom floors always clean and dry?	No Yes Do not know
19. Are there grab bars in all bathrooms?	No Yes Do not know
20. Is there supervision of older adults when they use the bathroom?	No Yes Do not know
21. Do the bathrooms have doors that open in both directions?	No Yes Do not know
22. Are assistive technologies available for older adults (e.g., walking support, food, hygiene, communication)?	No Yes Do not know
23. Is there a telephone or mobile phone available for older adults to use?	No Yes Do not know
24. Are there free water points for older adults?	No Yes Do not know
25. Are snacks available for older adults?	No Yes Do not know

## Appendix B

**Table A2:** Sociodemographic characterization of the study sample.

	Total
	*n* (%)
Sex	
Male	50 (28.9)
Female	123 (71.1)
Age group	
<40 years	65 (37.6)
40 to 50 years	48 (27.7)
51 to 60 years	31 (17.9)
>60 years	29 (16.8)
Health organization	
Primary Healthcare	129 (74.6)
Hospital Healthcare	44 (25.4)
Management position	
Primary Healthcare	
Coordinator	64 (37.0)
Technical council	56 (32.4)
Hospital Healthcare	
President	5 (2.9)
Clinical director	11 (6.4)
Director nurse	15 (8.7)
Executive member	9 (5.2)
Other	13 (7.5)
Total experience in management	
<3 years	83 (48.0)
4 to 6 years	39 (22.5)
7 to 9 years	15 (8.7)
10 to 12 years	16 (9.2)
>13 years	20 (11.6)

**Table A3:** Knowledge of literature concepts related to the Age-Friendly Principles.

	Total	
	*n* (%)	
	No	Yes
Age-Friendly Hospital	129 (74.6)	44 (25.4)
Age-Friendly Primary Healthcare	138 (79.8)	35 (20.2)
Age-Friendly Emergency Service	166 (96.0)	7 (4.0)
Age-Friendly Surgery Service	169 (97.7)	4 (2.3)
Age-Friendly Acute Care	170 (98.3)	3 (1.7)
Age-Friendly Health System	154 (89.0)	19 (11.0)
None of the above	60 (34.7)	113 (65.3)

**Table A4:** Knowledge of the Principles explained and the National Strategy for Active and Healthy Ageing 2017–2025.

	Total	
	*n* (%)	
	No	Yes
Knowledge of strategies to improve the training of professionals, the care system, and the physical environment	92 (53.2)	81 (46.8)
Know the measures proposed in the National Strategy for Active and Healthy Ageing 2017–2025	68 (39.2)	105 (60.7)

**Table A5:** Implementation of the general concept in health organizations.

	Total		
	*n* (%)		
	No	Yes	Do Not Know
The health organization develops an age-friendly policy that values and promotes the health, dignity, and participation of the older adults	42 (24.3)	100 (57.8)	31 (17.9)
The health organization’s strategic plan identifies compatibility with the older adults as a priority issue	75 (43.4)	67 (38.7)	31 (17.9)
The health organization has a team for the coordination and implementation of the Age-Friendly Principles	122 (70.5)	188 (10.4)	33 (19.1)
